# Targeting accuracy and impact of a community-identified waiver card scheme for primary care user fees in Afghanistan

**DOI:** 10.1186/1475-9276-9-28

**Published:** 2010-11-29

**Authors:** Laura C Steinhardt, David H Peters

**Affiliations:** 1 Department of International Health, Johns Hopkins Bloomberg School of Public Health, Baltimore, USA

## Abstract

**Background:**

User fees are a known common barrier to using health services, particularly among the poor. When fees are present, many facilities have waiver systems for poor patients to exempt them from paying. Targeting waivers to patients who need them most has been a challenge, especially in fragile states, where relevant data are limited and trust in institutions is low.

**Methods:**

Community-based targeting of vulnerable households was piloted in Afghanistan and evaluated for its feasibility, accuracy and effect on care-seeking. Waiver cards were distributed to very poor and female-headed households in catchment areas of 26 facilities in 10 provinces of Afghanistan in 2005 as one component of a larger health financing study. Households were nominated by community leaders using general guidelines to support 15% of the poorest members. In most cases, waiver cards were pro-actively distributed to them. Targeting accuracy, perceptions, as well the cards' effects on utilization were evaluated in 2007 through household surveys, health facility data, and in-depth interviews and focus group discussions with facility staff and community leaders.

**Results:**

The waiver system was implemented quickly at all but one facility charging fees. Facility staff and community leaders reported favorable perceptions of implementation and targeting accuracy.

However, an analysis of the asset index of beneficiaries indicated that although targeting was progressive, significant leakage and high levels of under-coverage occurred; 42% of cards were used by people in the wealthiest three quintiles, and only 19% of people in the poorest quintile received a card. Households with waiver cards reported higher rates of care-seeking for recent illnesses compared to those without cards (p = 0.02).

**Conclusions:**

Community identification of beneficiaries is feasible in a fragile state. Several recommendations are discussed to improve targeting accuracy of a waiver card system in the future, in light of this research and other international experiences.

## Background

Ensuring that social services and development programs are targeted to the poorest and most vulnerable people is a basic goal for increasing health and human capital, but one that has challenged governments, development institutions, and donors for decades. Targeting of benefits and services can be broad, or indirect, for example, through government spending on certain types of goods or services that are disproportionately consumed by the poor, or it can be narrow, whereby certain types of people, such as the poor, are targeted more directly [1]. Whereas broad targeting avoids the costs of identifying and verifying who should receive benefits, this approach can be expensive and may not reach the poorest groups [1]. Narrow targeting can have its own operational difficulties, reflecting tradeoffs between the advantages of focusing program benefits on those who need them most and the political, technical and financial difficulties of doing so [2]. This paper explores this inherent tradeoff between focused program benefits and targeting difficulties in the context of a community-identified waiver card scheme for user fees at primary health care facilities in Afghanistan.

### Types of 'narrow' targeting

Coady, Grosh, and Hoddinott identify three primary types of narrow targeting: 1) individual/household assessment, which can include verified means tests (e.g., collection of consumption data in low- and middle-income countries), proxy means tests, or community identification; 2) categorical targeting, whereby eligibility is defined by easily observable characteristics such as age, geography, gender, or other characteristics; and 3) self-selection, whereby poor households/individuals self-select to participate in a program whose opportunity costs (e.g., time) are too high or benefits too low (e.g., stigmatized) for wealthier households/individuals to participate [3].

Proxy means testing typically uses more easily collected information on household or individual characteristics, such as assets, housing characteristics, education, basic needs, food consumption, that correlate relatively well with welfare levels [2,4]. One challenge with proxy means tests involves how to score or translate proxy measures into poverty levels with meaningful cut-offs for programmatic decisions. Methods that have been used include simple summation of measures, weighted summation, with weights and cut-offs developed by expert consensus or face validity, or weights determined through a predictive algorithm such as regression on "gold standards" of poverty, such as per-capita or adjusted per-capita consumption [2,5]. Principal component analysis (PCA) on an index of durable assets and housing construction variables has recently proven useful for identifying relative wealth in a straightforward manner that appears to reflect underlying wealth levels relatively well [6]. Per capita or adjusted per capita expenditure or consumption are still widely considered the most reliable and valid method for assessing poverty, and these can provide absolute measures of poverty in reference to a minimum level of consumption/expenditure required to meet basic needs. Poverty assessments that use PCA are more useful for measuring relative poverty or wealth, though it can be argued that this is an equally relevant concept in development programs aiming to target the poorest members within a given community [7].

Community assessment, whereby community members or groups are involved in targeting activities, such as beneficiary identification or monitoring of benefits, can be applied to all three types of narrow targeting, but is most commonly used for individual/household assessments, whereby community groups or leaders act as the welfare agents in determining eligible beneficiaries at the local level. Potential benefits of community-based targeting include: lower costs, as expensive door-to-door surveys can be avoided and local agents typically do not need to be paid the same as educated bureaucrats; better information, as local agents may have a better sense of local poverty/vulnerability; and improved accountability, as there may be mutually reinforcing incentives between the local agent and community members to be honest [8]. The potential disadvantages of community-based assessment include poor capacity at local levels, elite capture, reinforcement of current unequal power structures, and the potential to create or enhance conflict/division within the community [8]. In Afghanistan, data at the individual household level on poverty are not readily available, and determinants of vulnerability are extremely variable at local levels, making it difficult to create standardized proxy means testing for poverty. The country has a rich tradition of community-based decision-making bodies, and several recent development programs, such as the National Solidarity Program, have attempted to create more representative and democratic community councils. In the Afghan context, community-based targeting may therefore be the most feasible way to identify households for benefits of social programs.

### Targeting in health

User fees and other types of cost sharing can decrease access to health care, particularly for poorer households, leading to under-utilization of beneficial services such as immunization [9]. Recognizing this, many countries have put in place waivers and exemptions for fees. Exemptions typically apply to certain types of services (e.g., delivery, tuberculosis treatment) or groups of patients (e.g., children under 5, pregnant women), and waivers exempt individual patients (e.g., poor patients) from fees [10]. Exemptions for certain underused services, such as deliveries, have become more common in recent years [11]. While exemption programs have met with some success [12], waiver programs have not had their intended effect. Waiver policies may not be standardized and tend to be at the discretion of health center staff, and inherent difficulties in targeting the poor in informal settings have rendered even well-intentioned policies largely ineffective [13-15]. As a result, some researchers have advocated for geographic targeting (i.e., granting waivers to entire geographic areas, based on their average socioeconomic conditions) over individual means testing [16], particularly with the advent of poverty mapping techniques allowing for small-area poverty and inequality statistics through household survey and census data [17,18].

However, recent pilot studies have provided some evidence that individual waivers through nomination by community groups can be effective in low-income settings where user fees are charged [19]. Health equity funds, whereby hospital fees and, in some cases, transportation and food expenses, for the poor are paid by a third party, have successfully involved health center management committees and local representatives/chiefs in identifying the poor in districts of Cambodia. Lao PDR has recently begun to pilot health equity funds based on the experience of Cambodia [20]. Aside from the relatively recent studies in Cambodia, well documented experiences of community-based targeting in the health sector in low-income settings are rare. Recent studies in Burkina Faso demonstrated the feasibility of community-based targeting for user fee waivers and free enrollment in community-based health insurance, but data validating the targeting effectiveness were not yet available [21,22]. The study presented in this paper uses data from Afghanistan to examine the targeting performance and effects on healthcare utilization of a community-identified waiver scheme in a very low-income, post-conflict setting. The primary objectives of this paper are: 1) to assess the feasibility and effectiveness of community nomination in targeting waiver cards to very poor and female-headed households; and 2) to evaluate the effects of waiver cards on utilization of curative care services.

## Methods

### Description of waiver card scheme in Afghanistan

As part of a health financing pilot study assessing different methods of community payment for health care, a standardized user fee system was piloted in summer 2005 at 27 facilities in 10 provinces of Afghanistan, including at six district hospitals, 13 comprehensive health centers, and eight basic health centers. Relatively small fees were charged at these pilot facilities for services (on average 5.5 Afghanis, equivalent to $0.11 US) and for any prescribed medications (average of 26% of the wholesale medication price). The cost of an average outpatient visit, including drugs, was a modest 11.5 Afghanis, or $0.23 US. Revenues were retained at facilities, and facility staff and community leaders (who together comprised the user fee committee) decided on which quality-improving measures (e.g., drugs to prevent stock-outs, infrastructure improvements, repairs, patient transportation, etc.) to spend the revenues. About 56% of the facilities had charged fees prior to participating in the pilot, but usually only for consultation, not drugs, and the revenues raised were not used at the facility but sent back to the regional primary care provider, in most cases a nongovernmental organization (NGO) contracted to deliver services in a given province. Additional details of the health financing pilot study are provided elsewhere [23,24].

Under the user fee pilot, waiver cards were distributed to very poor and female-headed households in the catchment areas of facilities implementing user fees. All members of a household receiving a waiver card were entitled to receive all services for free at the pilot facility. Preventive and promotive care, including immunization, ante-natal care, deliveries,

post-natal care and family planning, as well as emergency care and tuberculosis treatment, were free for all patients, including those without waiver cards. Facility staff and community leaders distributed information about the new user fees, exempt services, and waiver card system to community members in various ways they deemed to be most effective, including information dissemination at local bazaars, in mosques, at the facility itself, and through local community health councils.

Facility staff were trained on the waiver card system and given rough descriptive guidelines on characteristics of wealth groups, originally developed by experts carrying out a national poverty and vulnerability survey in 2003 [25]. Facility staff and user fee committee members were instructed to carry out enumeration of all catchment area households, and to work closely with community representatives (e.g., local village council *(shura*) members, *Mullahs*, and other village representatives) to have community leaders identify which households met the criteria for being female-headed or very poor. They were instructed to follow rough guidelines of 15% eligibility for waiver cards, according to the National Risk and Vulnerability Assessment, which found that 5-15% of households in a village are very poor on average [25]. However, the exact eligibility proportions, as well as specific details of how facilities should carry out the household enumeration, eligible household identification, and waiver card distribution, were left to individual facilities, given the local diversity of socioeconomic context and community participation and development structures. Data from the National Risk and Vulnerability Assessment survey conducted in 2003 revealed that estimates of food insecurity in the districts of the pilot health facilities ranged from 0% to 55.9%, with a mean of 21.8% of households rated as food insecure. Food insecurity data for each district were provided to health facility staff and community leaders.

One of the operational principles of the user fee pilot that was communicated to facility staff was that no one should be denied care due to inability to pay. Patients without waiver cards presenting at facilities who claimed they were unable to pay the user fees were initially questioned by the registrar. In these cases, the registrar was supposed to complete a screening questionnaire to determine if the patient was very poor and therefore eligible for a waiver card. The screening questionnaire template was developed by a committee within the Health Financing Department of the Ministry of Public Health, which included representatives from NGOs providing health services in each province. The template was based on ownership of certain assets, number of household members capable of working and who had worked in last six months, and recent income of the household, criteria that were loosely adapted from microcredit programs operating in Afghanistan. Facilities were urged to tailor the screening template to their local catchment areas.

### Data collection to evaluate waiver card scheme

As part of a larger evaluation of the health financing pilot study, a household survey was conducted in spring 2007--approximately one and a half years after pilot implementation--in the catchment areas of the health financing pilot facilities, including at 23 user fee pilot facilities, all in rural areas. Three user fee pilot facilities could not be surveyed for the evaluation due to poor security, and a fourth user fee pilot facility had not implemented the waiver card scheme, leaving 23 user fee pilot facilities that were included in the evaluation. At these facilities, two villages with greater than 100 households each and within 90 minutes' walking distance that had been randomly selected at baseline in 2004 were re-surveyed at follow-up, except in a few cases where villages had to be replaced due to poor security. The catchment areas were targeted for survey because there were no recent census data available at the time of the survey, and catchment areas represented households which were most likely to use the pilot facilities, as distance is inversely related to use of care [26]. Using a random start, up to 25 households were surveyed by selecting every second household [27] if they had a woman aged 18 or older with a child three years or younger, as the larger evaluation intended to measure child immunization rates, for a total of 50 households across two villages, per pilot facility. Between 50 and 150 households (mean = 120) in the catchment areas of user fee facilities were surveyed in each province, with the variation due to the number of operational user fee facilities in each province. The household survey included questions on illness, health care-seeking behavior, money spent on treatment, perceived quality of care at the pilot facility, knowledge of and perceptions about the waiver card system, including ownership of a waiver card, and household socioeconomic characteristics. All survey forms were edited for completeness and consistency and double-entered into a database in Kabul.

At follow-up, semi-structured qualitative interviews were conducted at 14 user fee pilot facilities. These facilities were purposefully selected to try and include both "typical" cases as well as more unusual cases (e.g., user fee facilities where the implementation process had been difficult). At each facility, up to three individual interviews were conducted with facility staff, along with one focus group interview with community members of the user fee committee, who were asked to come to the facility for the focus group, and one individual interview with a community leader from a surveyed catchment area village who was not a member of the user fee committee. Trained interviewers asked respondents about the waiver card scheme, including the targeting and card distribution process, among other aspects of the user fee pilot. Qualitative interviews were not tape-recorded due to cultural sensitivities but an additional surveyor took extensive hand-written notes during and after the interviews. Interviews were translated and then coded line-by-line in ATLAS.ti [28]. Codes relevant to the waiver cards were analyzed to identify common and outlying perceptions of facility staff and community leaders. Finally, quantitative data on the implementation process of the waiver card system were gathered through a systematic survey of the facility in-charge at each user fee facility. Ethical approval for the study was obtained from the Institutional Review Board of Johns Hopkins Bloomberg School of Public Health in Baltimore, Maryland, and from the Ethical Review Board in Kabul, Afghanistan. Informed consent was collected from all survey respondents and interviewees.

### Analysis methods

Principal component analysis (PCA) was run on a group of variables in the household survey including household ownership of 10 durable assets and housing characteristics, such as water source, lighting source, fuel source, and presence of toilet [6]. PCA was run separately for each of the 10 provinces, in order to ensure that measures of wealth were as specific as possible to the local context and with sufficient sample size. To increase the sample size for the PCA, households from additional facilities participating in the health financing pilot study surveyed during the follow-up evaluation that were randomized to free services (n = 9) or to serve as control facilities (n = 8) were included in the PCA for each province. (Household survey sample sizes were initially based on comparing study groups to one another and it was not possible to increase the number of households surveyed in user fee areas alone at follow-up.) The total number of households surveyed per province ranged from 124 to 373 (average = 242 households per province). The resulting household wealth scores from the follow-up survey were then used to divide households into five equal groups per province.

The following measures of accuracy and prediction errors, which are commonly used for assessing poverty prediction tools or performance of actual targeting [3,4,29], were used to evaluate targeting effectiveness:

- Under-coverage: actual poor wrongly classified as non-poor, as a proportion of the poor (equivalent to a false negative)

- Leakage: actual non-poor wrongly classified as poor, as a proportion of all beneficiaries (equivalent to a false positive)

- Progressivity ratio: proportion of total benefits going to poor as a proportion of population comprised by poor (a value of 1 indicates neutral targeting) [3], i.e. $\frac{\left(\#poorbeneficiaries/\#totalbeneficiaries\right)}{\left(\#poorhouseholds/\#totalhouseholds\right)}$

Alternative measures of wealth using PCA were created that also included variables on food insecurity and a household's measure of their perceived wealth, in addition to assets. Self reported (perceived) wealth was also used as a stand-alone proxy indicator for household wealth. In addition, PCA was run on all provinces combined ("national" PCA) and on just households in the user fee catchment areas, separately by province, to test the sensitivity of targeting performance to different specifications of the wealth index. The resulting wealth indices were fairly normally distributed within each province without major truncation or clustering of values.

Finally, the household survey data were used to analyze the effects of the waiver card on utilization of curative care services at the pilot facility, using the primary outcome of whether an individual household member sought care if ill in the past 30 days. Previous research has shown that participation in voluntary programs to reduce the cost of health care use, such as health insurance, may be related to underlying propensity to use health care [30-32], and this potential endogeneity may also apply to receiving a waiver card and needs to be taken into account [33]. Since selection into the waiver card scheme was not random, it could potentially be correlated with unobservable factors that affect individuals' or households' use of care (for example, propensity to value health or use care) and could therefore bias the results if not taken into consideration. Potential endogeneity and selection effects of the waiver card were assessed through several means. First, the Wu version of the Hausman test [34,35] assessed whether unobservable variables related to waiver card receipt were correlated with unobservable variables related to seeking care, by including the predicted values from a probit equation of card ownership in a probit model for the care-seeking equation, along with the potentially endogenous card variable. Next, we used a bivariate probit analysis, an appropriate analysis when a binary outcome (care-seeking, in this case) is determined by a binary variable of interest (receipt of waiver card) that is likely jointly determined with the outcome, necessitating a sort of simultaneous equations treatment [36,37]. We assessed whether rho, the correlation between the error terms of the two equations--one for care-seeking and one for waiver card--were significant, which indicates potential endogeneity that should be addressed in order to make valid inferences about causal effects.

There was little evidence of endogeneity of the waiver card and care-seeking (results available from authors upon request). Since unnecessary correction for endogeneity results in loss of efficiency, simple probit analyses are preferred in the absence of strong evidence of endogeneity. Therefore, a simple probit model was used for the care-seeking outcome, as follows:

Care-seeking* = ***X' β*1 **+ δ Card + ε

where Care-seeking* represents propensity to seek care; and ***X ***represents a vector of exogenous covariates that predict care-seeking, including: household demographic characteristics (the proportion of women of reproductive age in the household; proportion of children under five in the household; family size of 10 or more); household health (proportion of household members sick in the previous 30 days); socioeconomic factors (primary school or greater for the head of household; continuous wealth score; 1/wealth score^2^); women's care-seeking autonomy; province; type of nearest facility (hospital vs. clinic); average travel time to the facility; and individual characteristics, including age (under-five versus five and older), sex, and severity of illness (severe versus not severe). Women's care-seeking autonomy was measured by asking the household survey respondent (usually a woman) about which household member has the final say in deciding whether a sick child should be taken for care; responses of "mother/caretaker" were coded as one (23.3% of the sample), and other responses (e.g., father, mother-in-law, other) were coded as zero. Card is a binary variable that represents whether the household received a waiver card, and ε is a normally distributed error term.

Care-seeking models were also run for the poorest 40% of the sample, defined using the asset index wealth score, to assess the ultimate outcome of interest: the effect of the waiver card on use of care by the poor. All statistical analyses were adjusted for stratification by province and clustering by village using the survey commands in Stata Version 10.0.

## Results

### Waiver card implementation

Interviews with facility staff and community members of the user fee committee revealed that the household enumeration and waiver card distribution process was one of the more time-consuming and challenging aspects of the user fee pilot. The household enumeration process took two to three months, on average, and the waiver card distribution process another two to three months. Most facilities pro-actively distributed waiver cards, according to the pilot design, but slightly more than one-third stored cards at the facility for household members to retrieve. Facilities implemented the waiver card system in many different ways, drawing upon existing resources at the facility and within the community to identify households and distribute waiver cards, for example, using community health workers (CHWs) and local schoolteachers in some areas to enumerate households and identify eligible households.

Facility records indicated that cards were distributed to on average 12.2% of catchment area households (median = 10.8%; range = 4.1% to 23.4%). Typically, village representatives, including village council (*shura*) members, elders, the *Mullah/Imam*, as well as in some cases CHWs and other facility staff, were involved in nominating households for waiver cards, and the community members of the user fee committee usually had the final say about who received cards. At most facilities, 100% of identified households received a waiver card, but at four facilities, the user fee committee more carefully verified their eligibility (either with a door-to-door household survey or through less formal means), which resulted in only two-thirds to three-quarters of the initially nominated households actually receiving cards, as the remaining households were not considered to be very poor. Self-targeting still occurred to some extent, with patients without waiver cards presenting at facilities but still receiving services for free, as they claimed to be poor (and withstood initial questioning from the facility registrar). However, in many cases the registrar did not complete the screening questionnaire to determine waiver card eligibility and simply waived the fee if he/she believed that the patient truly could not pay. It was not always clear why the registrar did not complete the training form; at some facilities it appeared that insufficient attention to customizing the screening questionnaire to the local context meant that the register did not believe it accurately differentiated very poor households.

### Awareness of waiver card scheme and fees

Awareness of the fees at user fee facilities was high: only 1.3% of respondents in user fee catchment areas erroneously believed that services were free. Among those aware of fees, 94.7% knew that the facility charged a consultation fee, and 90.0% were aware of the charge for drugs. Regarding exempt services, there was higher awareness that immunization services were free (89.6%) compared to awareness of free reproductive health services (62.6% for ANC, 51.4% for family planning, and 40.5% for deliveries). Almost half the household survey respondents (48.6%) believed that the poor were exempt from paying fees, and 40.9% were aware that widows could receive services for free. However, nearly 40% of respondents (39.8%) were not aware that some patients were exempt from paying fees.

In the immediate catchment areas of user fee facilities, 40.3% of those who knew the facility charged fees were aware of the waiver card system. Those households in the catchment areas of primary care user fee facilities were more likely to have heard of the waiver card system (46.6%) compared to households in the catchment areas of user fee hospitals (17.9%), p = 0.001. Overall, 166 out of 1,148 households surveyed (14.5%) in catchment areas of user fee facilities with waiver card systems reported receiving a waiver card. A slightly greater proportion of households near primary care user fee facilities received cards (15.4%) compared to those near hospitals (11.2%), but this difference was not statistically significant.

### Targeting effectiveness

Households receiving waiver cards had lower average wealth scores from PCA than those without cards: -0.55 versus -0.09, p < 0.001, equivalent to about one-quarter of a standard deviation difference between the two groups (Table 1). They also had lower self-rated wealth scores and higher average food insecurity scores. Perhaps a more directly relevant measure for waiver card targeting is a household's ability to afford fees. Only 30.3% of respondents whose household had received a waiver card reported being able to pay fees with little to no difficulty, compared to 60.5% of those without a card, p < 0.001 (Table 1). Among those with waiver cards, 57.6% reported being unable to pay fees at all, versus only 17.9% of those without a card, p < 0.001.

**Table 1:** Characteristics of cardholders versus non-cardholders

	Card				p-value, difference
		
	No		Yes		
	***n = 982***		***n = 166***		

Any facility delivery among HH members in last 6 mos.	0.109	[0.088, 0.134]	0.212	[0.150, 0.291]	p = 0.001
Care sought if HH member ill in last 30 days^¥^	0.8719	[0.842, 0.897]	0.9569	[0.939, 0.970]	p < 0.001
Wealth status					
Average wealth score	-0.093	[-0.368, 0.182]	-0.547	[-0.809, -0.284]	p < 0.001
% in bottom 40%	0.434	[0.364, 0.507]	0.579	[0.494, 0.660]	p = 0.001
Average self-rated wealth score (1-5)*	2.129	[2.046, 2.213]	1.867	[1.735, 2.000]	p < 0.001
Average food insecurity score (1-5)**	1.892	[1.784, 2.000]	2.307	[2.115, 2.499]	p = 0.002
Can afford fees with little to no difficulty	0.605	[0.561, 0.647]	0.303	[0.232, 0.385]	p < 0.001
Mother/caretaker primary decision-maker in taking sick child for care	0.201	[0.171, 0.234]	0.428	[0.346, 0.514]	p < 0.001
Probability that HH member sick in last 30 days	0.197	[0.188, 0.208]	0.221	[0.194, 0.250]	NS
Average family size	7.05	[6.86, 7.25]	7.05	[6.787, 7.322]	NS
# kids <5	1.64	[1.58, 1.71]	1.60	[1.47, 1.74]	NS
# Women repro. age	1.42	[1.37, 1.47]	1.45	[1.35, 1.56]	NS
Female-headed household	0.032	[0.021, 0.048]	0.042	[0.017, 0.100]	NS
Any education, head of household	0.310	[0.267, 0.354]	0.259	[0.202, 0.316]	NS
Proportion of kids 5-18 in school	0.390	[0.020, 0.350]	0.398	[0.035, 0.328]	NS
Any participation in community forums	0.039	[0.026, 0.051]	0.030	[0.004, 0.056]	NS
Able to borrow from family or friends	0.755	[0.721,0.786]	0.758	[0.659,0.835]	NS
Unable to access any credit	0.080	[0.060,0.105]	0.085	[0.040,0.170]	NS
Average travel time, minutes	66.5	[51.2, 81.8]	65.8	[44.1, 87.5]	NS

Note: Average wealth score and % in bottom 40% based on PCA-derived basic asset index for each province, including free services and control facilities. Standard errors account for survey design. NS=Not Significant.

^¥ ^This outcome is measured at the individual household member level (n = 1,566 household members sick in last 30 days; 1,311 non-cardholders and 255 cardholders). All other variables measured at the household level.

* 1 = poorest; 5 = wealthiest

** 1 = low food insecurity; 5 = high food insecurity

In addition to differences in various measures of wealth and vulnerability, and differences in facility utilization (discussed below), a major difference between cardholders and non-cardholders pertained to "women's care-seeking autonomy". In cardholding households, the mother/caretaker was more than twice as likely to have the final say about taking a sick child for care than in non-cardholding households (42.8% vs. 20.1%, p < 0.001). Other than these specific differences, there were no significant differences between cardholders and non-cardholders in other characteristics, including demographics, illness rates, education, ability to borrow money, or other factors (Table 1).

**Figure 1:**
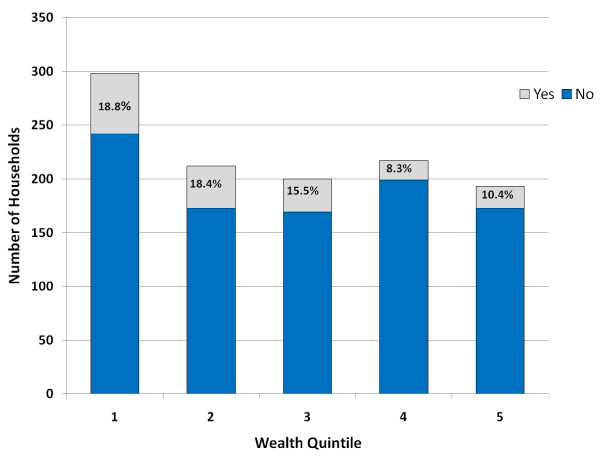
**Waiver card ownership, by wealth quintile**. Note: Wealth scores created from PCA on basic asset index and housing characteristics, for each province separately. 1 = Poorest; 5 = Least Poor.

There was extremely high correlation between wealth variables created with different combinations of assets (Pearson's correlation coefficient ranged from 0.97 to 0.99). As shown in Figure 1, 18.7% of the poorest quintile, and 18.4% of the second-poorest quintile received waiver cards, compared with an average of 11.3% in wealthier quintiles, when quintiles were created at the provincial level from wealth scores using the assets and housing characteristics only. Measures of leakage and under-coverage were calculated under two scenarios: first, considering as poor those with the lowest 20% of wealth scores; and second, those with the lowest 40%. Leakage was higher when the stricter cut-off of the lowest quintile was used to define the poor (65.9%), compared to a cut-off of the bottom two quintiles (42.1%) - see Table 2.

**Table 2:** Number and percent of households, by wealth quintile and card ownership, and selected measures of targeting performance

Wealth quintiles from PCA					Self-rated wealth status				
		**Card ownership**					**Card ownership**		

Quintile		No	Yes	Total	Rating		No	Yes	Total

1	N	242	56	298	1	N	233	56	289
	%	81.2	18.8	100.0		%	80.6	19.4	100.0

2	N	173	39	212	2	N	445	78	523
	%	81.6	18.4	100.0		%	85.1	14.9	100.0

3	N	169	31	200	3	N	253	30	283
	%	84.5	15.5	100.0		%	89.4	10.6	100.0

4	N	199	18	217	4	N	43	2	45
	%	91.7	8.3	100.0		%	95.6	4.4	100.0

5	N	173	20	193	5	N	7	0	7
	%	89.6	10.4	100.0		%	100.0	0.0	100.0

Total	N	956	164	1,120	Total	N	981	166	1,147
	%	85.4	14.6	100.0		%	85.5	14.5	100.0

Leakage - 20% poor				65.9%	Leakage - "1" = poor				66.2%
Leakage - 40% poor				42.1%	Leakage - "1" or "2" = poor				19.3%
Under-coverage - 20% poor				81.2%	Under-coverage - "1" = poor				80.6%
Under-coverage - 40% poor				81.4%	Under-coverage - "1" or "2" = poor				83.5%
Progressivity ratio 20% poor^1^				1.28	Progressivity ratio "1" = poor^1^				1.34
Progressivity ratio 40% poor^1^				1.27	Progressivity ratio "1" or "2" = poor^1^				1.14

^1 ^Progressivity ratio = proportion of total benefits going to poor/proportion of population comprised by poor (Coady et al. 2002). (A value of 1 indicates neutral targeting.)

Note: Wealth quintiles created from PCA on basic asset index for each province separately (including free services and control facilities). For quintile scores and self-rated wealth ratings, 1 = lowest; 5 = highest.

Under-coverage was similarly very high under both scenarios: 81.2% and 81.4%, respectively (see Table 2). Previous studies have found that the degree of under-coverage tends not to be sensitive to the poverty line cut-off, but that leakage rises dramatically with lower (more strict) poverty lines [2]. Results were similar using wealth quintiles created from scores using self-rated wealth and/or food security measures, in addition to assets (results not shown). However, leakage was considerably higher (54.9%) and under-coverage somewhat higher (85.0%) when considering the bottom 40% as poor and when quintiles were created at the provincial level from PCA on households only near user fee facilities. However, it is doubtful that provincial-level wealth scores from only the user fee areas are valid, as the sample size was only 50 or 100 in most provinces, too small for PCA to yield valid results [38]. Wealth quintiles created on all provinces combined indicated worse coverage and leakage results, as might be expected, since these do not reflect measures of local wealth (results not shown). When self-reported wealth status, measured on a scale from 1 (lowest) to 5 (highest) was used to assess these measures, under-coverage rates were similar but leakage declined to 19.3% when using a cut-off of a "1" or "2" rating as poor, as 70.8% of households rated their wealth status in the first two "quintiles", making it more likely that a card beneficiary would be "poor" (see Table 2). Among the 38 female-headed households (3.3% of all households) in user fee catchment area survey sample, only 7 (18.4%) received a waiver card, indicating ineffective targeting of female-headed households.

The progressivity ratio, defined as the proportion of benefits going to the poor compared to the proportion of the population comprised by the poor [3], was greater than one using PCA-determined poverty cut-off levels, as well as using the more subjective measure of self-rated wealth. The poor were 14-34% more likely to receive a waiver card compared to the non-poor (see Table 2). Results from the alternative PCA specifications were similar except for ratios calculated with wealth quintiles created from PCA on all provinces combined or from only the user fee facilities, which indicated more neutral targeting (results not shown).

#### Perceptions of waiver card targeting among facility staff and community leaders

Between two and seven community members of the user fee committee participated in the focus group interviews. In general, interviewed facility staff and community leaders reported that the waiver card system was implemented effectively, with little leakage but some under-coverage due to high rates of poverty in catchment areas. Facility staff and community leaders reported that in most areas, communities--involving some combination of village council (i.e. *shura) *members and elders, and, in some cases, CHWs and the *Mullah/Imam *as well--were authorized to draw up a list of very poor and female-headed households. The list was then typically approved by user fee committee members (both facility staff and community members) after verifying the economic status of the households through their collective knowledge and, in some cases, visits to the homes of potential card beneficiaries. Despite initial problems at some facilities, especially those with no previous fees, related to many people wanting cards and claiming to be poor, the process ultimately proceeded smoothly at most facilities and was transparent and effective in targeting deserving households, according to facility staff. Some facility staff mentioned that in order to be more transparent, either a survey had to be conducted of all households in the catchment area, or the *Mullah/Imam *should be more involved in the process, as he generally knows the economic status of his followers well. A few facility staff raised the issue of needing continual or updated assessments, for example to identify people moving into the area who might need a waiver card, or to give cards to/take cards from households whose economic status had changed since the last assessment. In several cases, community leaders remarked that the number of cards should be increased, as not all very poor households received a card due to limited numbers. Two of the 13 community leaders interviewed mentioned that they felt "family and tribal" pressure to distribute cards to those they knew, although both these leaders mentioned they resisted this pressure successfully, as one leader noted below.

I: Was [the waiver card nomination and distribution] process fair and just?

R: Yes, it was very just.

I: Did you face any problem in this process?

R: Yes. I went to a house and there a person who told me that her sister is poor and should be given an exemption card. When I went there, his sister was neither poor nor a widow. He first was insisting and even was ready to fight but later I tried and convinced him and then he agreed.

#### - Community leader, catchment area village of user fee District Hospital

One of the community representatives on the user fee committee at this same facility remarked that the targeting was generally good, although not perfect, alluding to the difficulty of complete transparency in local allocation systems:

R: We are representatives of people and we always have been in favor of justice during the distribution of the cards. Those who are tasked this work, they might have been able to do their work around 70% and it is possible that there would be some people who are not poor and have acquaintance with the representatives but have received the cards. And also there would be some people who are really poor but have not received the exemption cards. We as elders of the community have tried to provide the cards to those who really deserve it. But it is not possible to implement any project in the villages with 100% justice.

#### - Community leader involved in user fee committee, catchment area village of user fee District Hospital

Households were asked an open-ended question as part of the household survey about their general perceptions of the waiver card system. Among those respondents who were aware about the waiver card system (292 of 982 non-cardholders (29.7%) and 100% of 166 cardholders), 68.5% of non-cardholders and 84.3% of cardholders made generally positive comments, including statements like "it has good effects on people", "it makes going to the clinic easy", and "it helps poor people solve their problems". Twenty-eight percent of non-cardholders and 8% of cardholders thought that more cards overall were needed, making statements such as, "additional households need cards", and "cards should be given to all households" or noting that they themselves wanted a card. Only 4.8% of non-cardholders and 2.4% of cardholders made statements about the unjustness of the targeting system, such as "cards are given to those who know one another", "they don't give cards to poor people," or "there should be a re-assessment to identify poor households".

### Effects of waiver cards on utilization of curative care services

Most cardholders reported that their household used both more curative (85.5%) and more preventive care (83.7%) as a result of the waiver card, although 11.3% and 13.2% reported using less curative and preventive care, respectively, as a result of the card, and 3.1% reported no change. Overall, 95.7% of sick individuals from cardholding households compared with 87.2% of sick members of non-cardholding households sought care when ill in the last 30 days with an illness they deemed worthy of seeking care, p < 0.001. Adjusted for other factors, the probit model indicated that individuals from cardholding households were 3.1 percentage points more likely to seek care than sick members of households without cards, p = 0.016 (Table 3). In addition to geographic location, age less than five years and presence of a severe (vs. mild or moderate) illness, were significantly related to seeking care, as was women's care-seeking autonomy (Table 3). Education of the head of household also had a positive effect on care-seeking, although this was only marginally statistically significant (p = 0.066). Probit analysis of the poorest 40% of the sample indicated that the waiver card had an even stronger effect, increasing care-seeking by 6.2 percentage points, p < 0.001 (results not shown).

**Table 3:** Marginal effects from probit model of care-seeking

	Probit Model		
	**CARE-SEEKING**		
	**dy/dx**	**SE**	**p-value**

Card	0.0306	0.0127	0.016 ++
Prop. women repro. Age	0.1426	0.0811	0.079 +
Prop. kids <5	-0.0061	0.0485	0.900
Family size >9 members	-0.0018	0.0140	0.896
Women's careseeking autonomy	0.0245	0.0108	0.023 ++
Prop. sick HH members last 30 days	-0.0689	0.0311	0.027--
Female-headed household	0.0190	0.0270	0.482
More than 6 years education, head of HH	0.0285	0.0155	0.066 +
Wealth	0.0039	0.0044	0.373
1/Wealth^2^	0.0002	0.0001	0.041 ++
Province (reference = Kapisa)			
Parwan	0.0388	0.0161	0.016 ++
Wardak	0.0385	0.0138	0.005 +++
Samangan	0.0270	0.0184	0.143
Balkh	-0.0485	0.0350	0.165
Badghis	0.0127	0.0216	0.557
Farah	-0.0068	0.0312	0.827
Nimruz	0.0115	0.0235	0.624
Sari-Pol	0.0672	0.0198	0.001 +++
Panjshir	0.0614	0.0184	0.001 +++
In catchment of hospital (ref. = catchment clinic)	0.0009	0.0136	0.947
Avg. travel time, hours	-0.0152	0.0083	0.067 -
Age < 5 years	0.0297	0.0127	0.020 ++
Severe illness (vs. mild/moderate)	0.0332	0.0133	0.012 ++
Female	-0.0094	0.0103	0.363

N	1486		

+ = p < 0.10; ++ = p < 0.05; +++ = p < 0.01; same meaning for negatively associated variables.

Standard errors adjusted for cluster sampling.

Despite elimination of the financial barrier at the pilot facility for cardholders, there still remained financial and other types of barriers to accessing care for households with and without waiver cards. Overall, more than half (58.0%) of household respondents reported facing a problem in getting health care that they or someone in the household needed in the past year. This was slightly higher among cardholders (64.5%) compared to non-cardholders (57.1%), though the difference was not statistically significant. The largest reported barrier was lack of money for treatment, reported by nearly half (49.5%) of respondents (see Table 4). Interestingly, slightly more cardholders (53.6%) than non-cardholders (48.8%) reported that lack of money for treatment was one of the barriers to accessing care they faced in the last year, though this difference was not statistically significant. Lack of transportation (24.0% overall) and lack of money for transport (20.6% overall) were other important barriers. The only statistically significant difference in reported barriers to accessing needed care by cardholders versus non-cardholders was the availability of someone to take a child for treatment: 28.9% versus 14.8%, p < 0.001.

**Table 4:** Reported barriers to accessing care in the last year, by card status (%, [95% CI])

	Card					
		
	No		Yes		Total	
No money for treatment	48.8%	[43.6%, 54.0%]	53.6%	[44.0%, 63.0%]	49.5%	[44.8%, 54.1%]
No transport available	24.0%	[19.9%, 28.7%]	24.1%	[18.2%, 31.2%]	24.0%	[20.1%, 28.4%]
No money for transport	19.9%	[15.7%,24.9%]	24.7%	[18.3%, 32.4%]	20.6%	[16.5%, 25.3%]
No one to take child for care	14.8%	[12.2%, 17.8%]	28.9%***	[21.8%, 37.2%]	16.8%	[14.4%, 19.5%]
No drugs available	13.8%	[10.8%, 17.6%]	15.7%	[9.5%, 24.7%]	14.1%	[11.0%, 17.9%]
Health center too far	12.7%	[8.8%, 18.1%]	9.6%	[5.3%, 16.8%]	12.3%	[8.6%, 17.2%]
No one to accompany female	8.0%	[5.6%, 11.3%]	6.0%	[3.0%, 11.6%]	7.8%	[5.6%, 10.7%]
Inconvenient hours of provider	4.2%	[2.7%, 6.3%]	3.0%	[ 1.5%, 6.0%]	4.0%	[2.7%, 6.0%]

Source: Household survey. Standard errors adjusted for cluster sampling.

*** p < 0.001

## Discussion

From the perspective of community leaders and facility staff, the waiver card system was able to be implemented successfully in Afghanistan in 26 of 27 facilities across 10 provinces piloting the user fee system under the health financing pilot study. Given the flexibility built into the pilot design, as well as substantial local heterogeneity in local community structures in Afghanistan [39], the waiver card system implementation process was very context-specific and heterogeneous. Waiver cards were distributed to households within one year of pilot implementation and, in most cases, within six months, a relatively short time frame given the lack of additional human and financial resources devoted to the waiver card scheme. However, despite training and continued technical assistance for implementation, this lack of dedicated staff and resources made the waiver card system one of the more time-consuming and challenging aspects of the user fee pilot. Households in the catchment areas of pilot facilities had high awareness of fees, but relatively low awareness of the waiver card system (40.3%) and of exempt preventive/promotive services, indicating incomplete community mobilization and information dissemination.

Facility staff and community leaders involved in the user fee and waiver card systems reported that the targeting process was fair, transparent, and relatively accurate. However, they noted in many cases that more cards were needed as there were not enough to cover the large numbers of poor households in the area, and some mentioned occasional occurrences of leakage to wealthier households. It is important to keep in mind the perspectives and potential biases of the facility staff and village leaders, who were directly involved in the waiver card process and therefore have reason to present themselves and the process as fair and effective. Although there was limited information available to capture household's viewpoints, open-ended survey questions of card-holding and non-cardholding households indicated that they had mostly favorable perceptions of the targeting process among those aware of the waiver card system.

Household survey results using wealth scores derived from PCA on household assets revealed that although the card distribution was overall pro-poor, in the sense that the poor had a higher probably of receiving a card, there was significant under-coverage and leakage, a finding similar to that for the *Kartu Sehat *(health card) program for the poor in Indonesia [3,33], where leakage and under-coverage were estimated to be 25.5% and 69.7%, respectively, considering the poorest 40% as "poor" (Authors' own calculations based on [33]). Even in Cambodia, which has largely been considered an example of successful targeting, one evaluation four years after the initial targeting found under-coverage and leakage to be about 40% each, reflecting imperfections in targeting and the dynamic nature of poverty over time [40]. The goal is not necessarily perfect targeting, and efforts to increase the precision of targeting can have large costs as well, not only in terms of actual costs to more accurately target only the poor, but in terms of political economy as well, as broad-based support for the program across income groups may collapse and undermine the benefit, known as the "paradox of targeting" [8,30,41]. Part of the large under-coverage in Afghanistan may have been due to the suggested 15% eligibility for waiver cards. Although facilities and communities were instructed that this was flexible, they may have used this guide too stringently, leading to under-coverage in poorer areas. Interviews from some facilities also revealed that facility staff allowed community leaders to nominate households in each village, but capped the proportion of households in each village to be uniform across the catchment area, even though some villages are poorer than others, further exacerbating under-coverage problems. It was not clear why targeting of female-headed households did not seem to be effective.

A major strength of the waiver card system was pre-identification of households in their communities. Certain health equity funds operating in Cambodia have shown that eligibility screening and identification of poor patients after they reach the hospital results in low awareness of the fund among the target population and continued financial barriers to access among those unaware [42,43]. However, compared to other pre-identification schemes documented in the literature, this pre-identification process was much less thorough and involved, and only four of the 25 facilities expended efforts to systematically verify the eligibility of nominated households through household visits. Among health equity funds in Cambodia that use pre-identification, although their exact processes for determining beneficiaries varies, nearly all followed up on initial lists of households gathered from government census and from village leaders to systematically evaluate nominated households in each village, and used dedicated staff and explicit scoring of proxy means tests (e.g., demographic and housing characteristics, education of family members, and ownership of various household assets as well as other factors) [44]. Researchers working on evaluating health equity funds in Cambodia have recommended that pre-identification not be done only once but through a series of steps to evaluate the eligibility of households [44].

It appears that possession of a waiver card led to increased use of services among households, and this effect was even more pronounced for the poor, increasing their care-seeking by 6.2 percentage points. Additional analyses (not shown) indicated that it is likely that the card also led to increased use of delivery services at the facility, although it was not possible to tease apart this effect with accuracy due to relatively low use of delivery services overall and the limited sample size of the household survey. Even though increases in delivery use associated with the card were modest, at just over 3 percentage points, according to the simple probit model, this represents a 33% increase in delivery use compared to non-cardholders, an important gain in a country with high maternal mortality ratios and very low rates of institutional delivery [45,46]. The findings are also relevant for other demand-side interventions and demonstrate the potential for mechanisms such as conditional cash transfers or other demand-side subsidies to increase use of underutilized services (e.g., delivery, immunizations), as these interventions can be even more comprehensive in addressing financial barriers to care and, in some cases, nonfinancial barriers such as transportation availability.

Cardholders also reported greater care-seeking autonomy among women. While this may reflect selection bias related to these households' propensity to value health and use care, it is also possible that ownership of the waiver card increased the autonomy of women in these households, as they no longer needed to consider the cost implications of seeking care as seriously and did not need to rely as much on the male household authority figures to give them money for user fees. Previous studies from low- and middle-income countries have found that women typically have the responsibility for taking sick children for care, but that men tend to control cash in the household [47]. However, it is not possible to determine with certainty whether waiver cards were given to households with greater women's care-seeking autonomy, or whether they actually helped increase care-seeking autonomy among receiving households, as this variable was not measured at baseline.

The increase in service use from the waiver card is consistent with findings from Cambodia, where equity certificates for pre-identified households led to hospitalization rates among beneficiaries that were one-and-a-half to eight times greater than non-health-equity-fund recipients, and with findings from an evaluation of the Health Care Fund for the Poor in Vietnam [48-51]. Similar results were found in Indonesia, following distribution of health cards to vulnerable households to help counteract the Indonesian economic crisis [33].

### Limitations

Several important limitations apply to the results from this study. First, it is important to keep in mind that comparisons for the purposes of evaluating targeting accuracy were done without a strong measure of household consumption for comparison. Although they have been shown to be valid indicators of underlying wealth, proxy scores from asset indices created from PCA are just that: proxies. They may better reflect accumulated wealth than more liquid types of cash or assets that can be used to pay facility fees. The PCA methodology is most useful for identifying relative wealth, and not absolute wealth, such as wealth in relation to a poverty line created from household consumption. However, since the PCA was run separately for each province, the wealth scores should reflect local measures of relative wealth that are also important in targeting. Future research on targeting in Afghanistan should consider using both relative and absolute measures of wealth, as well as capturing more in-depth perspectives on wealth and targeting from community members.

In addition, although we did not find strong evidence of endogeneity of the card with the care-seeking outcome, we cannot rule out this possibility and determine definitively whether the card led to increased use of services. Evidence points in that direction, but without baseline data from the same households, it is difficult to conclude with certainty that the waiver card led to increased care-seeking.

### Lessons learned and policy implications

If Afghanistan is to re-implement the waiver card system in the future, for example as a formal mechanism to increase access by the poor to services at higher-level hospitals, which still charge fees, what lessons can it learn from this pilot experience and those of its neighbors? Several targeting-related and operational changes should be considered.

#### Targeting considerations

First, Afghanistan should build upon the success it has had in implementing a community-nominated waiver card system that was perceived by community leaders and facility staff as relatively transparent and fair. This is an important accomplishment in a post-conflict setting, where trust in institutions, including at the community level, related to publicly financed services, has been worn down from years of fighting and ethnic rivalries. Involvement of community members in beneficiary nomination provides a way to increase community participation in health and contributes to rebuilding trust in institutions at the community level, a particularly important benefit in post-conflict settings [19].

As suggested in several interviews with facility staff and community leaders, a new waiver card system should consider more active involvement in nominating households by *Mullahs/Imams*, who are widely respected in communities and tend to have good knowledge of individual household conditions. Religious leaders, viewed as honest and trustworthy, have successfully been involved in community-based targeting in Cambodia, and researchers have pointed to the community-based management of targeting, including high involvement of the pagodas and their trusted volunteers and monks, as one of the important keys to success of the equity fund [19].

Another contributing factor to the successful community-based targeting used in several Cambodian health equity funds is the presence of clearly defined eligibility criteria [19], which were much looser in Afghanistan, given the wide geographic variability of the pilots and the absence of cash income in many areas. Although the individual NGOs implementing health equity funds in different regions of Cambodia ended up using different proxy means targeting criteria from one another, researchers have identified the relatively uniform social and demographic conditions in Cambodia as a key factor in the success of health equity funds [52]. Afghanistan is much more diverse geographically, culturally, and socioeconomically than Cambodia. This indicates that a more diverse targeting strategy, including geographic targeting for high-poverty districts where the cost of individual household targeting does not make sense, may be warranted. This approach has been successfully implemented in Vietnam, which uses a mix of individual characteristic and geographical targeting to identify beneficiaries for its Health Care Fund for the Poor [51].

Despite the geographic, socioeconomic, and cultural diversity in Afghanistan, which may preclude the development of a uniform national set of targeting criteria, specific verifiable criteria should still be developed for future targeting schemes in Afghanistan. This will make eligibility easier to verify and the process more transparent. Similar to what is currently being piloted in Lao PDR, the process could begin in a similar manner as before with a broader list of poor households provided by village leaders, using pre-identification criteria, and then screened by dedicated staff or trained volunteers using the objective criteria [20]. The objective criteria could--and certainly would be expected to--vary by geographic location. Close coordination with existing sources of data on poverty and vulnerability in Afghanistan, such as the biennial

National Risk and Vulnerability Assessment (NRVA) survey, could prove extremely valuable for implementation of successful geographic targeting and development of localized objectively verifiable proxy indicators for community targeting. It would also be worthwhile to pursue collaboration with other sectors, such as agriculture, livelihoods, education, and others, to examine the feasibility of developing a common waiver card or targeting scheme across multiple sectors where targeting can be an important aspect of service delivery.

#### Operational implications

The waiver card scheme was positively viewed by both community leaders and facility staff, despite the lack of reimbursement to facilities for the foregone user fee revenues. This reimbursement of foregone user fee revenues is one factor that has been cited as key to the success of the health equity funds in Cambodia [42,49,53] as well as for health financing programs for the poor in Vietnam [48]. Even if fee revenues do not constitute a significant portion of staff income in Afghanistan, as they do in Cambodia and Vietnam [52], a waiver card scheme, especially at the hospital level where inpatient stays represent larger sources of revenue, should consider directly reimbursing facilities for use of care by cardholding patients.

Additionally, if waiver cards are used for hospital fees, it will be important to reimburse for indirect costs as well, including transportation, food, and other costs associated with seeking care [51]. This research indicated that financial access still appears to be a barrier, even among cardholding households, and waiver card programs should be expanded to cover additional costs of seeking care. It is noteworthy that there was no evidence of "unofficial" charges to waiver cardholders at user fee facilities, but the reported financial barriers may have been due to other costs related to care-seeking, such as transportation fees, food, lodging, or seeking care at other providers (e.g., private providers). However, even more generous reimbursement packages may not be sufficient to remove all financial barriers to seeking care, as research in Cambodia found that health equity fund beneficiaries still were unable to afford the upfront costs of seeking hospital care [43].

Finally, it is critical to continue to invest in monitoring and evaluation efforts if a revised form of waiver cards is implemented in the future. This research contributes to the relatively scarce literature on community-identified targeting, demonstrating that it is possible to implement a pro-poor and favorably viewed waiver card scheme within a relatively short time and with limited additional support. However, targeting did not perform well according to PCA-derived relative wealth scores, indicating that these measures may not be the most appropriate for capturing disposable income and/or that community leaders are taking other important factors into consideration when nominating households.

The post-conflict setting in which the scheme was carried out provides encouraging evidence that beneficial health services delivery strategies can also contribute to community participation and rebuilding of institutions at the local level. These results lend support to the notion that waiver cards can increase utilization of care, even for services that have multiple barriers to use. Afghanistan abolished user fees for primary care in 2008, citing the results of the larger pilot study in which the waiver card scheme took place, but barriers to access remain. Further research should examine the effects of demand-side financing interventions to improve utilization of important public health services in Afghanistan, as well as a waiver card scheme for costly hospital-level services to prevent catastrophic expenditures and encourage use, building on the initial results from this study.

## Competing interests

The authors declare that they have no competing interests.

## Contributions

LS conceived of the study, performed the statistical analyses, and prepared the initial draft of the manuscript. DP is the PI of the larger study, led its design and coordinated data collection and assisted with drafting of the manuscript. Both authors read and approved the final manuscript.
